# Supplementary material for: Ecological differences among hydrothermal vent symbioses may drive contrasting patterns of symbiont population differentiation
Source: mSystems. 2023 Jul 26;8(4):e00284-23. doi: 10.1128/msystems.00284-23 (PMC10469979; doi:10.1128/msystems.00284-23)
Supplement: Supplemental Material — Supplemental figures, table legends, methods, and results. [file msystems.00284-23-s0001.pdf]

Supplementary Material for

Ecological differences among hydrothermal vent symbioses may drive contrasting patterns of symbiont population differentiation

Corinna Breusing*, Yao Xiao, Shelbi L. Russell, Russell B. Corbett-Detig, Sixuan Li, Jin Sun, Chong Chen, Yi Lan, Pei-Yuan Qian, Roxanne A. Beinart*

***Email:** corinnabreusing@gmail.com, rbeinart@uri.edu

This PDF file includes:

Figures S1 to S4
Legends for Tables S1 to S11
Supplementary Methods and Results
References

Other supplementary materials for this manuscript include the following:

Tables S1 to S11

Supplementary Figures

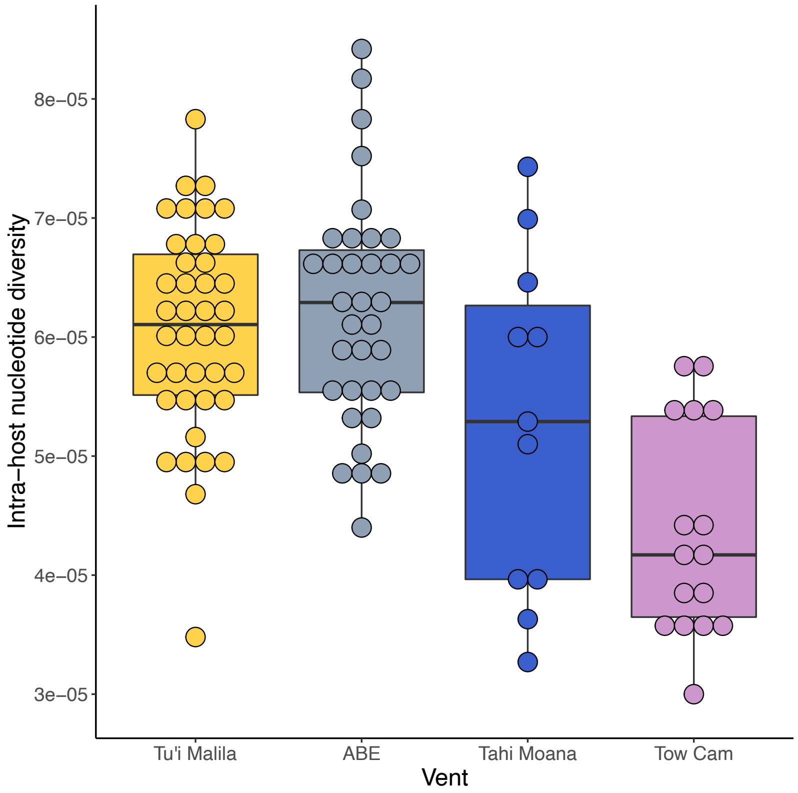

Fig. S1 Intra-host nucleotide diversities (π) at each vent site. Mean π values are significantly lower at Tow Cam than at ABE and Tu'i Malila based on pairwise t-tests ($p < 0.000002$).

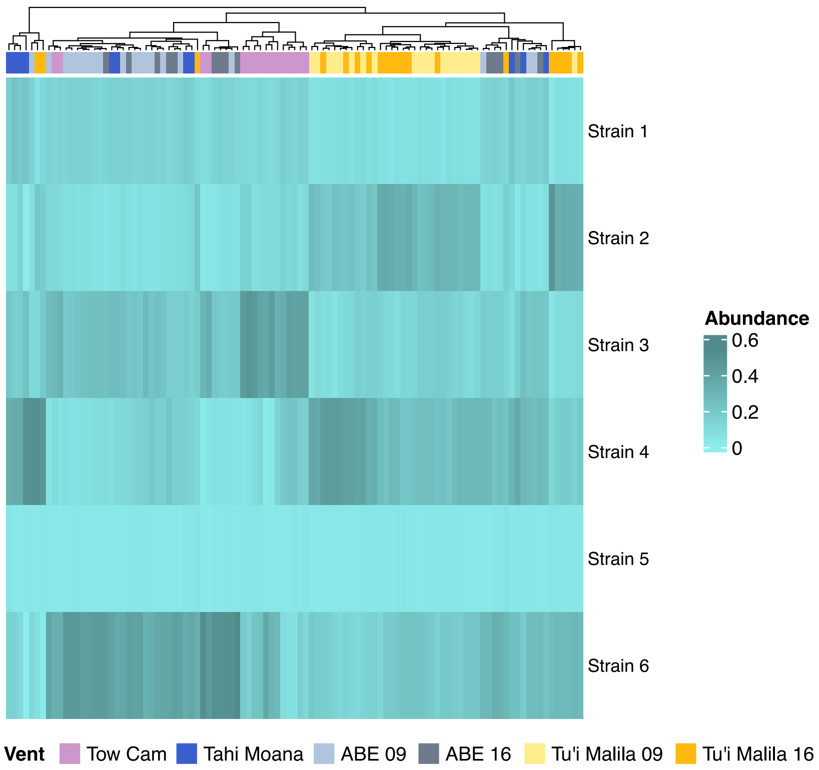

Fig. S2 Strain composition inferred through haplotype extraction from metagenomic core variant co-occurrence with DESMAN. Although DESMAN detects notably less strains than the BAYESPATHS algorithm, a similar pattern of strain abundance clustering by vent field, in particular for Tow Cam and Tu'i Malila, is observed with this method.

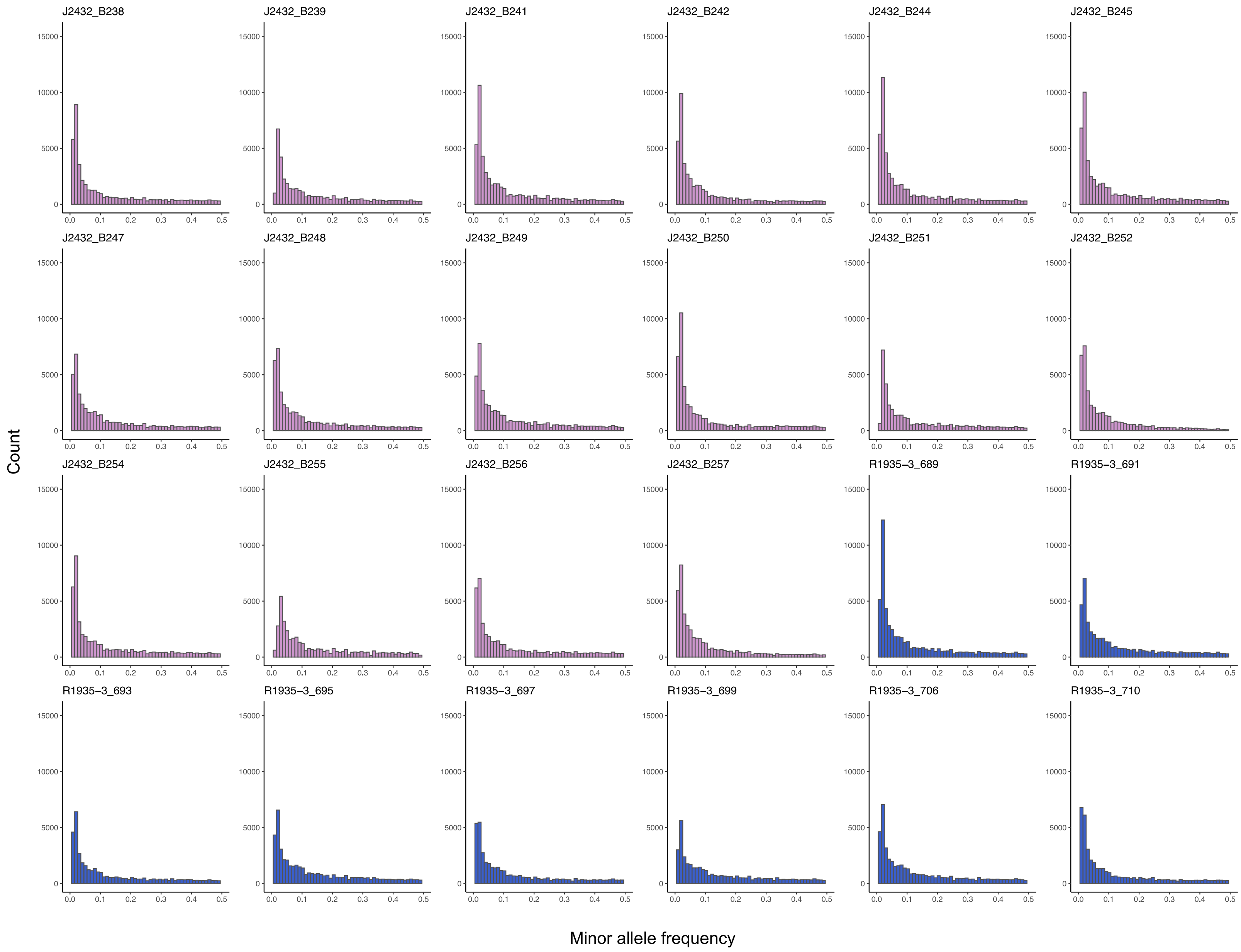
J2432_B238
J2432_B239
J2432_B241
J2432_B242
J2432_B244
J2432_B245
J2432_B247
J2432_B248
J2432_B249
J2432_B250
J2432_B251
J2432_B252
J2432_B254
J2432_B255
J2432_B256
J2432_B257
R1935−3_689
R1935−3_691
R1935−3_693
R1935−3_695
R1935−3_697
R1935−3_699
R1935−3_706
R1935−3_710
Count
Minor allele frequency

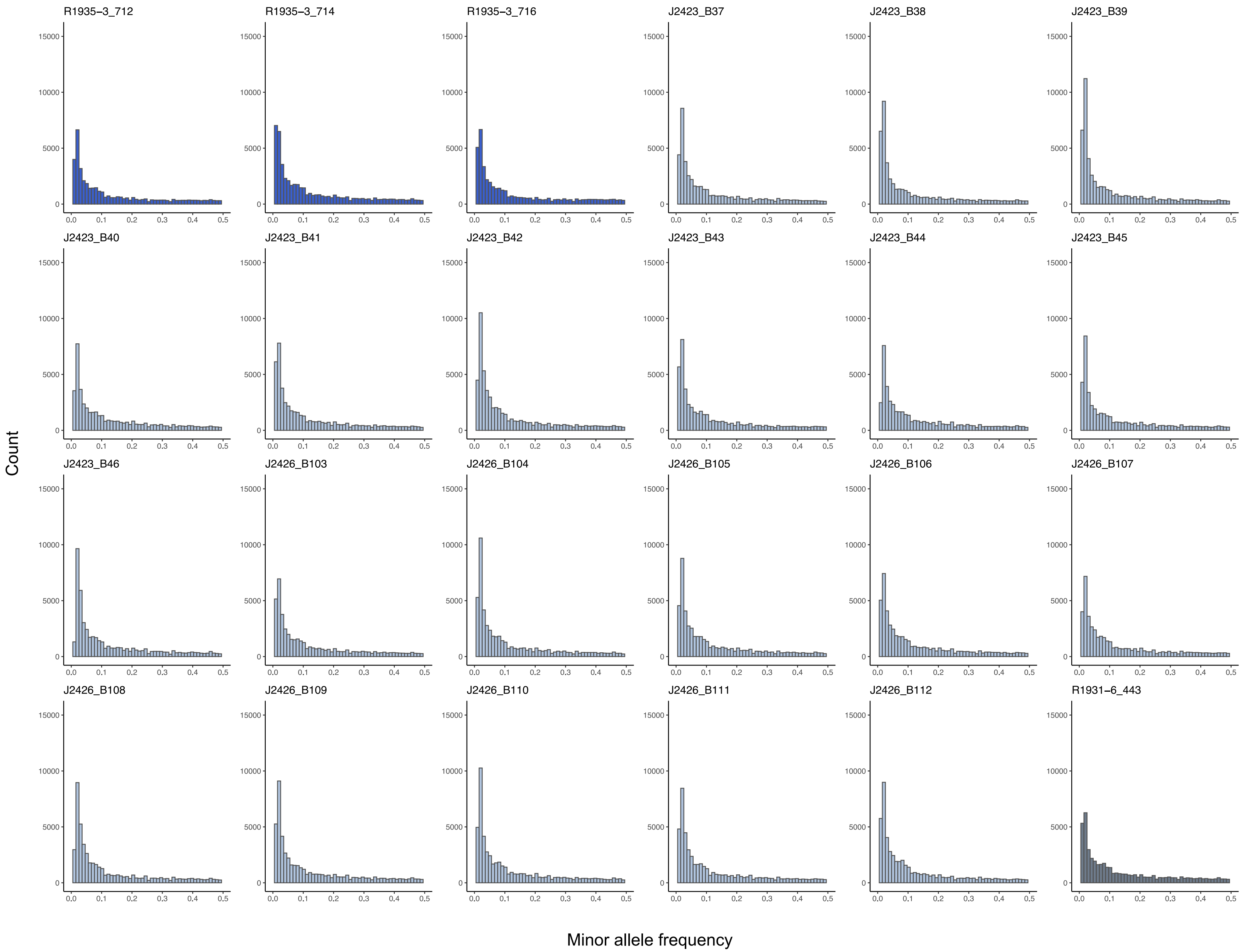
R1935–3_712
R1935–3_714
R1935–3_716
J2423_B37
J2423_B38
J2423_B39
J2423_B40
J2423_B41
J2423_B42
J2423_B43
J2423_B44
J2423_B45
J2423_B46
J2426_B103
J2426_B104
J2426_B105
J2426_B106
J2426_B107
J2426_B108
J2426_B109
J2426_B110
J2426_B111
J2426_B112
R1931–6_443
Count
0
5000
10000
15000
0.0
0.1
0.2
0.3
0.4
0.5
Minor allele frequency

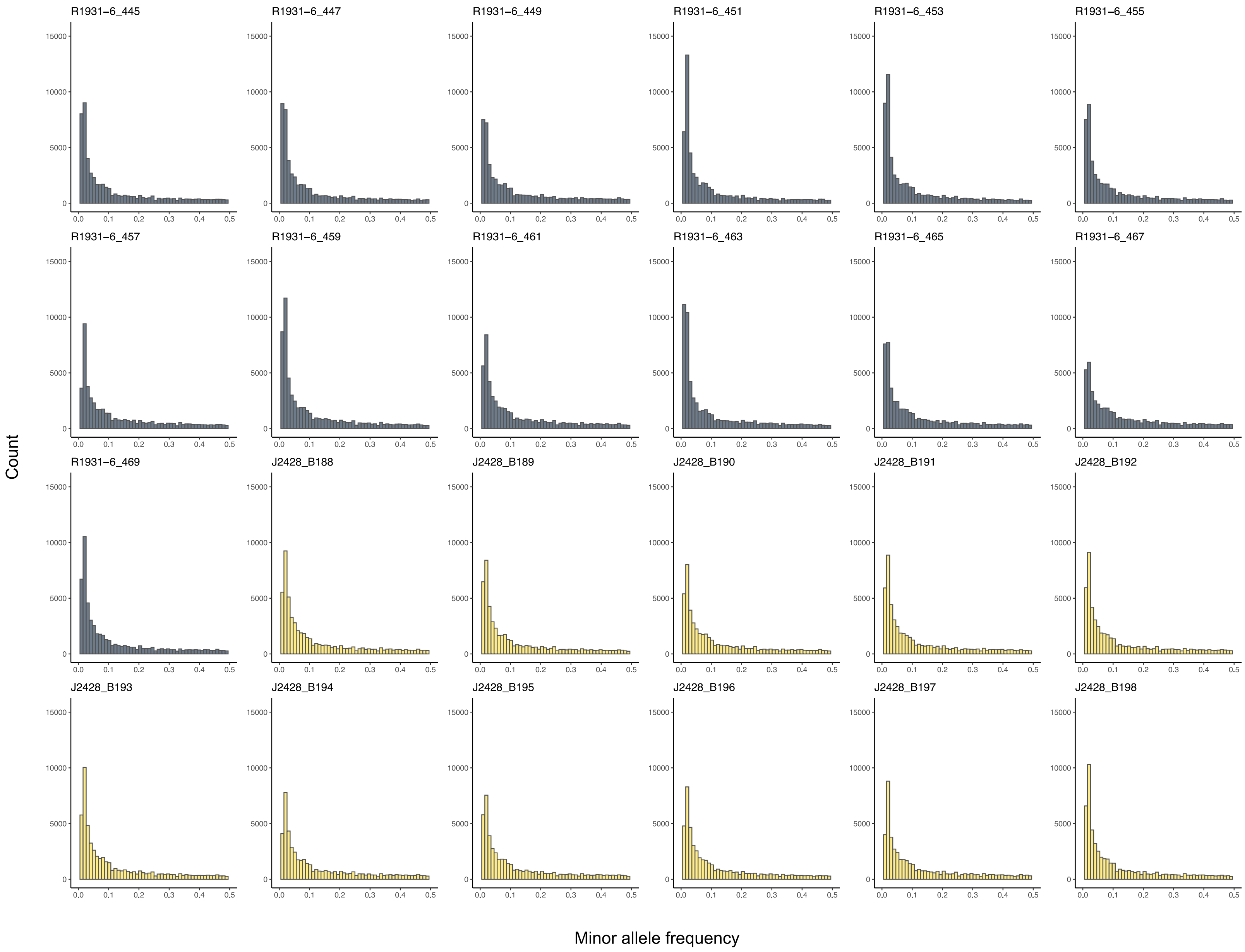

R1931−6_445
R1931−6_447
R1931−6_449
R1931−6_451
R1931−6_453
R1931−6_455
R1931−6_457
R1931−6_459
R1931−6_461
R1931−6_463
R1931−6_465
R1931−6_467
R1931−6_469
J2428_B188
J2428_B189
J2428_B190
J2428_B191
J2428_B192
J2428_B193
J2428_B194
J2428_B195
J2428_B196
J2428_B197
J2428_B198
Count
Minor allele frequency

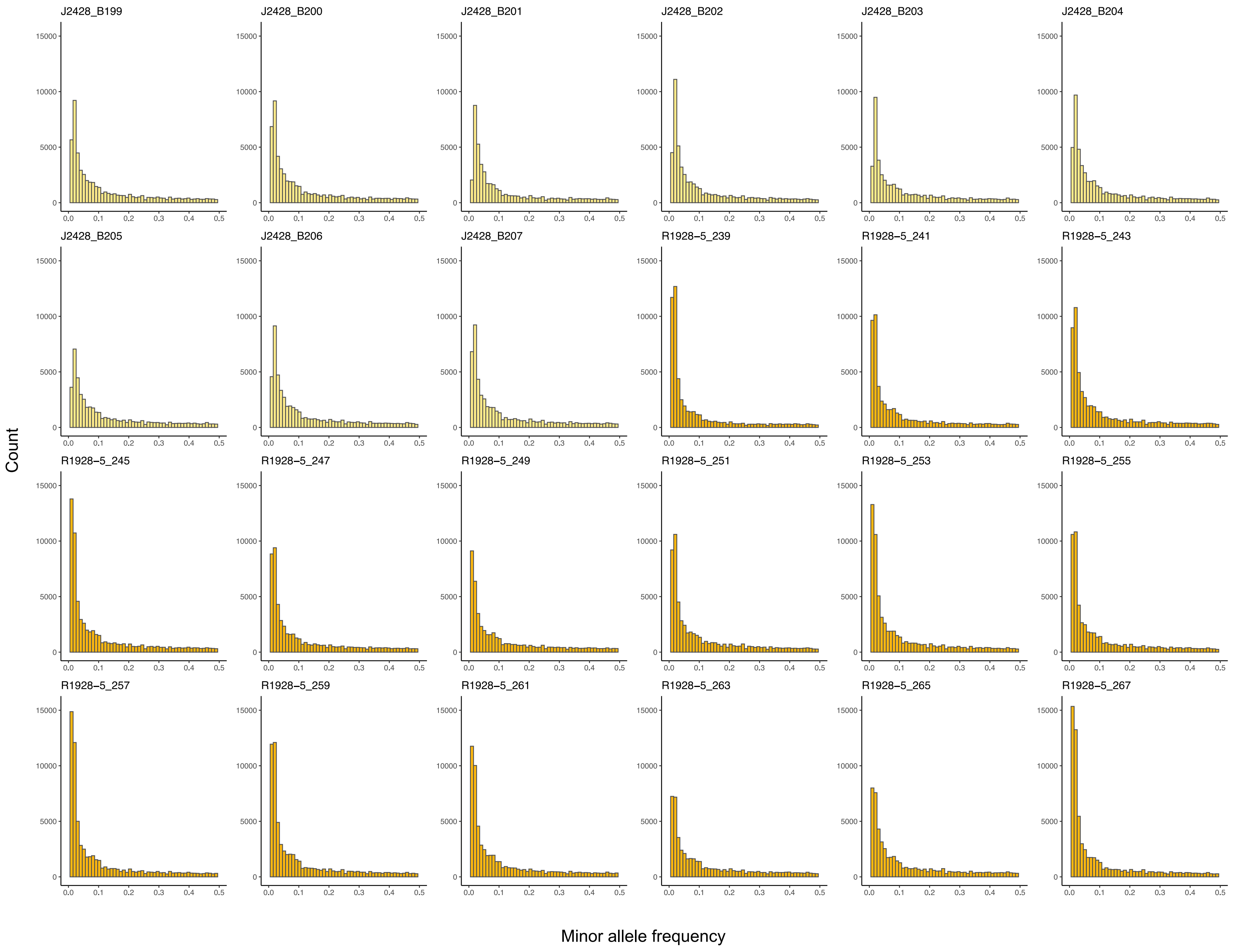
J2428_B199
J2428_B200
J2428_B201
J2428_B202
J2428_B203
J2428_B204
J2428_B205
J2428_B206
J2428_B207
R1928−5_239
R1928−5_241
R1928−5_243
R1928−5_245
R1928−5_247
R1928−5_249
R1928−5_251
R1928−5_253
R1928−5_255
R1928−5_257
R1928−5_259
R1928−5_261
R1928−5_263
R1928−5_265
R1928−5_267
Count
Minor allele frequency

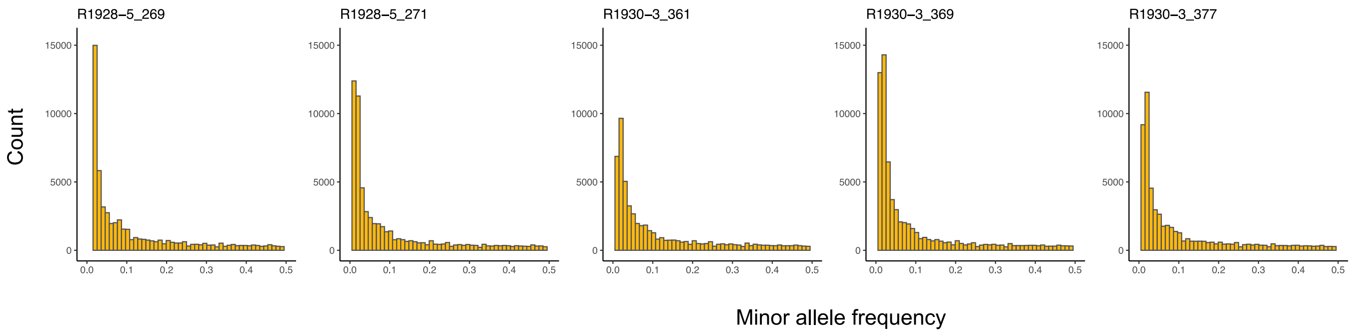

Fig. S3 Minor allele frequency spectra for each mussel sample. AFSs follow expectations for an equilibrium neutrally-evolving population, in accordance with the high genetic diversity observed for *Ca.* T. endoseptemdiera and other mussel symbionts as previously described [1].

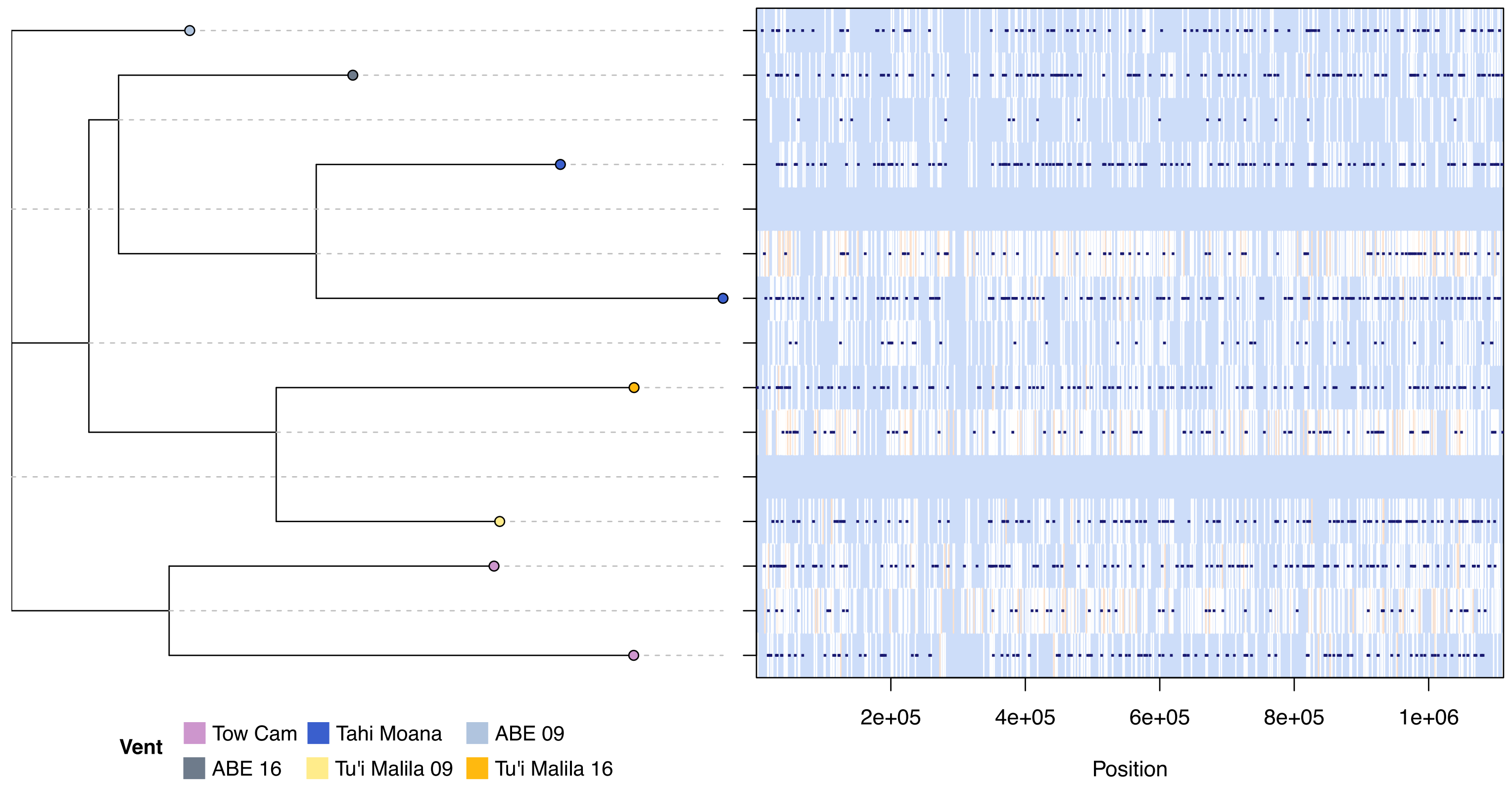

Fig. S4 Analysis of genomic recombination in *Ca.* Thiodubiliella endoseptemdiera with ClonalFrameML. Left: recombination-corrected phylogeny, with tip colors indicating the isolation source of the symbiont genome. Right: recombination and mutation events across the core genome. Recombination events are shown as midnightblue bars, while mutation events are indicated as white to red bars, with white denoting non-homoplasic substitutions and increasing redness denoting increasing levels of homoplasy. Light purple bars indicate absence of substitutions. Recombination is pervasive across the core genome of *Ca.* Thiodubiliella endoseptemdiera, though rates are likely underestimated due to repeated recombination events at any given genomic position.

Supplementary Table Legends

Table S1 Collection information for *Bathymodiolus septemdierum* samples in the Lau Basin.

Table S2 Effects of methodology and vent habitat on symbiont genetic variation as assessed through PERMANOVAs. PERMANOVAs were performed with the adonis2 function in R.

Table S3 Overview of the *Bathymodiolus septemdierum* transcriptome assembly.

Table S4 Quality statistics and taxonomic assignments for metagenome-assembled genomes of *Ca*. Thiodubiliella endoseptemdiera.

Table S5 Overview of the *Ca*. Thiodubiliella endoseptemdiera pangenome assembly.

Table S6 Pairwise F_{ST}s (lower diagonal) and P_{ST}s (upper diagonal) between symbiont populations of individual hosts.

Table S7 Strain abundances in analyzed mussel hosts. Abundance values represent normalized coverages as reported by STRONG. Non-normalized strain coverages are given in brackets.

Table S8 Detected mitochondrial variants in each sample. Variants were determined across the whole mitochondrial genome including and excluding the control region, which is typically difficult to assemble and can therefore lead to false variant calls.

Table S9 Differentially preserved genes between *Ca*. Thiodubiliella endoseptemdiera populations from Tu'i Malila, ABE, Tahi Moana and Tow Cam. For each vent location the proportion of symbiont samples containing the respective gene variant is shown.

Table S10 Local (vent site-specific) recombination analyses with RHOMETA within each symbiont species. ρ/θ = recombination to mutation rate ratio, r/m = relative effects of recombination to mutation.

Table S11 Results from *Ca.* Thiodubiliella endoseptemdiera 16S rRNA sequence searches in IMNGS at a 100% identity cutoff. Potential hits are found at different locations in the Western and Eastern Pacific Ocean. The name of the representative OTU target sequence includes the SRA run, a sample read number included in OTU construction and the OTU cluster size.

Supplementary Methods and Results

Full length 16S rRNA reference sequences of *Ca*. T. endoseptemdiera were uploaded to the IMNGS (Integrated Microbial Next Generation Sequencing) website [2] and processed using the Parallel Similarity search function with a similarity threshold of 99%. Only database entries with 100% identity to the reference sequence were included for further analysis. IMNGS is a freely available platform that integrates all 16S rRNA gene amplicon datasets available in the Sequence Read Archive and *de novo* constructs dataset-specific OTU clusters at a 97% threshold for sequence search and analysis [2]. The representative sequence of each OTU cluster is used as the target sequence to align with the query sequence [2].

Our analyses suggest that the 16S rRNA sequence of *Ca*. T. endoseptemdiera matches at 100% identity with the constructed OTU sequences of three different BioProjects (PRJNA275905, PRJNA275901, PRJNA382809) that performed 16S gene amplicon profiling at methane seeps along Hydrate Ridge off the Oregon coast, an East Tropical Northern Pacific oxygen minimum zone and coral reefs around the Fiji Solomon Islands (Table S11). These results indicate that *Ca.* T. endoseptemdiera might be broadly distributed in the Pacific Ocean. However, the V4 hypervariable region is typically a poor discriminator among different *Bathymodiolus* symbiont species so that further genomic analyses will be needed to confirm the presence of *Ca*. T. endoseptemdiera at the identified locations.

References

1. Russell SL, Pepper-Tunick E, Svedberg J, Byrne A, Ruelas Castillo J, Vollmers C, et al. Horizontal transmission and recombination maintain forever young bacterial symbiont genomes. *PLoS Genet* 2020; **16**: e1008935.
2. Lagkouvardos I, Joseph D, Kapfhammer M, Giritli S, Horn M, Haller D, et al. IMNGS: A comprehensive open resource of processed 16S rRNA microbial profiles for ecology and diversity studies. *Sci Rep* 2016; **6**: 33721.
